# Structural and functional frontal‐executive dysfunction in patients with isolated REM sleep behaviour disorder

**DOI:** 10.1002/alz.095216

**Published:** 2025-01-09

**Authors:** Micaela Mitolo, Luca Baldelli, Giovanni Sighinolfi, Luisa Sambati, David Neil Manners, Raffaele Lodi, Federica Provini, Caterina Tonon

**Affiliations:** ^1^ IRCCS Istituto delle Scienze Neurologiche di Bologna, Bologna, Italy Italy; ^2^ University of Parma, Parma Italy; ^3^ IRCCS Istituto delle scienze neurologiche di Bologna, Bologna, Italy Italy; ^4^ IRCCS Istituto delle Scienze Neurologiche di Bologna, Bologna, Bologna Italy; ^5^ University of Bologna, Bologna, Bologna Italy; ^6^ University of Bologna, Bologna Italy

## Abstract

**Background:**

Isolated REM Sleep Behavior Disorder (iRBD) is a well‐recognized prodromal state of an underlying α‐synucleinopathy, occurring several years before an overt neurodegenerative disorder becomes fully manifest. iRBD has been related to poorer cognitive performance and higher frequency of mild cognitive impairment (MCI). The aim of this study was to explore in detail, with structural and functional MRI, frontal‐executive dysfunction in iRBD patients and to evaluate its association with cognitive performance.

**Method:**

Thirty‐two iRBD patients (24 males; age, mean±SD = 67.9±7.1 years) and thirty age‐matched healthy controls were recruited and underwent an extensive neuropsychological assessment and multiparametric MR acquisition. The MR protocol (3T) included T1‐w volumetric (isotropic 1mm^3^), diffusion weighted imaging (b = 2000 s/mm^2^, 64 directions) and resting‐state (TR = 0.735s, 10 minutes) acquisitions. Brain volumetry analysis was conducted using FreeSurfer. TBSS was applied to evaluate white matter microstructural alterations. Resting state networks were investigated with ICA. Correlations between MR parameters and neuropsychological variables were explored with Spearman’s test.

**Result:**

iRBD patients, when compared with healthy controls, showed worse performance on tests related to attentive‐executive, memory, and visuospatial functions (i.e., cancellation test p = 0.041, similarities p = 0.015, 15‐words recall p = 0.004, and simple drawing copy p = 0.007); they also showed volume reduction in left rostral anterior cingulate (p = 0.005). TBSS highlighted increased anterior corpus callosum and forceps minor Radial Diffusivity in iRBD patients (p <0.05). Significant correlations were found between this alteration and executive dysfunctions (i.e. Stroop test, r = 0.46, p = 0.009). Moreover, fMRI data showed ‘Executive control’ network alteration in iRBD patients within cortical (i.e. superior and middle frontal gyrus, opercular cortex, paracingulate and precentral gyrus) and subcortical structures (i.e. putamen and caudate); these data correlated with worse performance in executive‐attentive tests (i.e., cancellation time, r = ‐0.45, p = 0.018 and frontal assessment battery, r = 0.44, p = 0.023).

**Conclusion:**

This study showed that structural and functional frontal‐executive alterations in iRBD patients are associated with attentive‐executive functioning, findings that are similar to those observed in some synucleinopathies. Further studies with bigger samples and follow‐ups are needed to confirm these results, and to monitor the trajectory of these alterations longitudinally.

**Acknowledgement**: This study was supported by the Italian Ministry of Health (#GR‐2019‐12369242).

